# Incidence of Cardiovascular Instability in Patients With Guillain-Barré Syndrome: A Retrospective Study

**DOI:** 10.7759/cureus.52778

**Published:** 2024-01-23

**Authors:** Noel James, Sanjeeva Reddy, Uma Maheshwari, Natarajan Elamurugan, Nirumal Kumar, Arun N Kumar, Sathya D Tejaswini, Lakshmi Narasimhan Ranganathan, Balasubramanian S

**Affiliations:** 1 Institute of Neurology, Madras Medical College, Chennai, IND

**Keywords:** lower cranial nerve palsy, liver function tests, onset to admission time, egris score, cardiovascular instability, guillain-barre syndrome

## Abstract

Introduction

Guillain-Barré syndrome (GBS) is an autoimmune disease affecting radicles and peripheral nerves resulting in acute flaccid paralysis. Respiratory failure, autonomic dysfunction, and secondary complications such as pneumonia, and venous thromboembolism are the major causes of death and disability in GBS. Cardiovascular complications play a major role in the prognosis of GBS patients. The aim is to determine the incidence of cardiovascular instability in GBS patients and to see if there are any specific risk groups associated with the development of cardiovascular instability.

Methodology

This is a retrospective descriptive study conducted in a tertiary care center in South India. Data on 50 consecutive GBS patients were collected from hospital records including case sheets, death summaries, and discharge summaries. Patients with evidence of sepsis, blood loss, heavy alcohol consumption, and chronic liver disease were excluded from the study. Baseline demographic data, symptom onset to admission time, baseline Erasmus Guillain-Barré Syndrome Respiratory Insufficiency Score (EGRIS), and baseline liver function tests were documented. The presence of heart rate and blood pressure fluctuations was noted from the records. Frequency data were calculated from the categorical variables. Analysis of non-parametric variables by chi-square test was done using IBM SPSS Statistics for Windows, Version 25.0 (Released 2017; IBM Corp., Armonk, New York, United States).

Results

Cardiovascular instability was present in 15 (30%) patients in the study population. It was present in all patients (100%) who require mechanical ventilation. The incidence of cardiovascular instability was higher in patients who had lesser onset to admission times (41.9% vs 10.5%; p=0.019), EGRIS≥4 (40.6% vs 11.1%; p=0.029), and lower cranial nerve involvement (40% vs 6.7%; p=0.018).

Conclusion

Of patients with GBS, 30% developed cardiovascular instability during their disease course. Patients with lesser onset to admission times, EGRIS ≥4, and those with lower cranial nerve involvement had a greater incidence of cardiovascular instability.

## Introduction

Guillain-Barré syndrome (GBS) is one of the most common causes of acute flaccid paralysis. It is characterized by symmetric areflexic paralysis and is associated with albumin-cytologic dissociation. It occurs worldwide with an incidence of about one to two for every 100,000 people. The incidence of GBS is increasing worldwide. There is a trend for higher incidences among older age groups and the incidence is slightly higher in males [1]. Up to 20% of GBS patients remain severely disabled and the mortality rate is 5%. There are multiple variants of GBS with both axonal and demyelinating patterns in nerve conduction studies.

GBS is usually preceded by an infectious event in most of the cases. Molecular mimicry is the postulated mechanism in which antibodies formed against pathogens exhibit cross-reactivity with gangliosides found in myelin sheath [2]. The most frequent infection identified as a trigger for GBS is that of *Campylobacter jejuni*, seen in about 25-50% of adults with GBS, with a higher prevalence noted among Asian countries [3]. Other infections that are associated with GBS are cytomegalovirus, Epstein-Barr virus, influenza A virus, *Mycoplasma pneumoniae*, and *Haemophilus influenzae* [4,5].

The diagnosis of GBS requires that a clinical nadir should be reached within four weeks and that there may be no more than two treatment-related fluctuations. The clinical presentation of GBS is variable with multiple variants: classic sensorimotor variant, pure sensory variant, pure motor variant, bifacial palsy with paresthesia, paraplegic variant, pharyngeal-cervical-brachial (PCB) variant, dysautonomia variant, ataxic variant. In addition, there are GQ1b spectrum disorders namely the peripheral nervous system predominant Miller Fisher syndrome and the central nervous system predominant variant, Bickerstaff brainstem encephalitis [6].

The clinical course of GBS and prognosis are also highly variable. One-third of all patients remain ambulant though with weakness throughout the entire course of illness [7]. Others develop life-threatening respiratory muscle weakness or bulbar weakness of dysautonomia, necessitating mechanical ventilatory assistance. Despite optimal treatment with immunotherapy, the mortality rate is around 5%.

There are various situations where ICU admission has been recommended including progressing respiratory muscle weakness, evolving respiratory distress, bulbar dysfunction, dysphagia, dysautonomia, and Erasmus Guillain-Barré Syndrome Respiratory Insufficiency Score (EGRIS) > 4 [8]. Most often, GBS is preceded by an infectious event, most commonly a diarrheal illness. Upper respiratory tract infection has also been associated with the occurrence of GBS. However, upper respiratory tract infections have been associated with a better prognosis as compared to those that are preceded by a diarrheal illness [9].

Autonomic dysfunction with features such as bladder dysfunction, constipation, tachycardia, and hypertension are also poor prognostic factors in GBS [10]. Autonomic dysfunction has been reported in two-thirds of the population, predominantly in the form of cardiac dysregulation [11]. There have also been some observations that abnormal liver function tests (LFTs) at presentation have been associated with a poor prognosis [12]. We aim to study the incidence of cardiovascular instability among patients with GBS and explore the possible association of elevated LFTs, onset to admission time, and EGRIS at admission with cardiovascular instability among patients with GBS.

## Materials and methods

This study is a retrospective descriptive study on GBS patients who were admitted during 2021-2022 at Madras Medical College, Chennai, India. Patients diagnosed to have GBS based on the Asbury clinical criteria comprising (i) progressive and symmetric weakness, (ii) hyporeflexia or areflexia, (iii) disease course of less than four weeks along with electrodiagnostic features of demyelination or axonopathy fitting into Uncini criteria. Patients with significant alcohol intake (>21 drinks/week for men and >14 drinks/week for women), a history of chronic liver disease, evidence of sepsis (fever, lung consolidation, and rising total leukocyte count), and those with evidence of blood loss were excluded.

Data in the records concerning clinical examination of patients with testing for cranial nerve dysfunction was noted. The presence/absence of facial nerve palsy and bulbar dysfunction was noted and findings documented and the presence of facial nerve palsy or IX, X cranial nerve palsy was taken as lower cranial nerve dysfunction. Muscle power testing was noted and graded according to the Modified Research Council (MRC) scale. Time from onset of symptoms and the day of admission was documented and further stratified as ≤5 days from onset and ≥6 days from onset. LFTs (SGOT (serum glutamic-oxaloacetic transaminase) and SGPT (serum glutamic pyruvic transaminase)) done at admission were noted and stratified as ≤2 times upper limit of normal (ULN) and ≥3 times ULN.

The presence of documentation of labile blood pressure (or) labile heart rates (or) unexplained arrhythmias was taken as evidence of cardiovascular instability. Blood pressure lability is defined as fluctuation >20% documented at least twice in a day at an interval greater than one hour and heart rate fluctuations are taken as heart rate variability >10% documented at least twice in a day at an interval of greater than one hour. Unexplained arrhythmia includes sinus bradycardia, atrial fibrillation, flutter, and ventricular tachycardia. EGRISs calculated at admission were noted. Baseline LFTs taken at the time of admission were further stratified as multiples of the upper limit of normal. The incidence of autonomic dysfunction amidst the various strata of elevated or normal LFTs (≤2 times of ULN and ≥3 times of ULN), EGRIS (≤3 and ≥4), and symptom onset to admission time (≤5 days and ≥6 days) were recorded.

Association between the various strata of LFTs, EGRIS, and symptom onset to admission time was done using chi-square non-parametric test. IBM SPSS Statistics for Windows, Version 25.0 (Released 2017; IBM Corp., Armonk, New York, United States) was used for the analysis of data.

## Results

Data from 50 consecutive patients were collected in the study as per protocol. The mean age of the study population was 43.9 years (SD 16.92). The mean duration from symptom onset to admission is 5.5 days (SD 2.68) and the mean MRC score for motor strength is 34.08 (SD 8.26). Out of the 50 patients, 34 (68%) were male and 16 (32%) were female. The onset to admission time was less than or equal to five days for 31 (62%) patients, and 19 patients (38%) were admitted after five days from symptom onset. Elevation of LFTs of ≥3 times the ULN was noted in eight (16%) patients. Cardiovascular instability was noted in 15 (30%) of cases. Eight patients (16%) required mechanical ventilation due to respiratory failure during their hospital stay. Lower cranial nerve involvement (VII, IX, X cranial nerves) was present in 35 (70%) patients in the study group. Out of the 50 patients, six (12%) succumbed to the illness. Electrophysiological evaluation revealed acute inflammatory demyelinating polyneuropathy (AIDP) (60%) to be the most frequent variant, followed by acute motor and sensory axonal neuropathy (AMSAN) (28%), and acute motor axonal neuropathy (AMAN) (12%) (Table 1).

**Table 1:** Demographic variables of the study population (N=50) ULN: upper limit of normal; SGOT: serum glutamic-oxaloacetic transaminase; SGPT: serum glutamic pyruvic transaminase; AIDP: acute inflammatory demyelinating polyneuropathy; AMAN: acute motor axonal neuropathy; AMSAN: acute motor sensory axonal neuropathy

Parameter		Frequency (percentage)
Sex	Male	34 (68%)
	Female	16 (32%)
Onset to admission time	≤5 days	31 (62%)
	≥6 days	19 (38%)
Liver function tests (SGOT/SGPT)	≤2 times ULN	42 (84%)
	≥3 times ULN	8 (16%)
Cardiovascular instability	Present	15 (30%)
	Absent	35 (70%)
Requirement for mechanical ventilation	Yes	8 (16%)
	No	42 (84%)
Lower cranial nerve (VII, IX, X) involvement	Yes	35 (70%)
	No	15 (30%)
Outcome	Alive	44 (88%)
	Death	6 (12%)
Electrophysiologic type	AIDP	30 (60%)
	AMAN	6 (12%)
	AMSAN	14 (28%)

Patients who have a shorter symptom onset-admission time have a greater relative frequency of developing cardiovascular instability. Of the 31 patients who were admitted within the first five days, 13 (41.9%) patients had autonomic disturbances, whereas of the 19 patients who got admitted after five days of symptom onset, only two (10.5%) developed cardiovascular instability. The association between earlier onset to admission time and cardiovascular instability was statistically significant with a p-value of 0.019 (Table 2).

**Table 2:** Frequency of cardiovascular instability in patients with different onset to admission times in Guillain-Barre syndrome Data has been represented has % of total, p< 0.05 has been considered as significant

Symptom onset to admission time	Cardiovascular instability		Total	Chi-square	p-value
	Yes, n (%)	No, n (%)			
≤ 5 days	13 (41.9%)	18 (58.1%)	31	5.534	0.019
≥ 6 days	2 (10.5%)	17 (89.5%)	19		

The relative incidence of cardiovascular instability was higher among the eight patients with LFT values at admission ≥ 3 times ULN. Of the patients in this subgroup, 50% (n=4) had cardiovascular instability. On the other hand, among the 42 patients with LFT levels ≤ 2 times ULN, only 26.2% (n=11) had cardiovascular instability. However, the correlation did not attain statistical significance (Table 3).

**Table 3:** Frequency of cardiovascular instability in patients with normal and elevated liver function tests Data has been represented has % of total, p< 0.05 has been considered as significant

Liver function tests	Cardiovascular instability		Total	Chi-square	p-value
	Yes, n (%)	No, n (%)			
≤ 2 times of upper limit of normal	11 (26.2%)	31 (73.8%)	42	1.814	0.178
≥ 3 times of upper limit of normal	4 (50%)	4 (50%)	8		

The incidence of cardiovascular instability was higher in those with EGRIS ≥ 4. Of the 32 patients with an EGRIS ≥4, 40.6% (n=13) had evidence of cardiovascular instability whereas 11.1% (n=2) of the 18 patients with EGRIS ≤3 had evidence of cardiovascular instability. The association was statistically significant with a p-value of 0.029 (Table 4).

**Table 4:** Frequency of cardiovascular instability in patients with high and low EGRISs Data has been represented has % of total, p< 0.05 has been considered as significant EGRIS: Erasmus Guillain–Barré Syndrome Respiratory Insufficiency Score

EGRIS	Cardiovascular instability		Total	Chi-square	p-value
	Yes, n (%)	No, n (%)			
EGRIS ≤ 3	2 (11.1%)	16 (88.9%)	18	4.778	0.029
EGRIS ≥ 4	13 (40.6%)	19 (59.4%)	32		

Lower cranial nerve dysfunction is considered present if the patient has either VII cranial nerve or IX, X cranial nerve palsy. The proportional occurrence of cardiovascular instability was higher in patients with lower cranial dysfunction. Of the 35 patients with lower cranial nerve dysfunction, 40% (n=14) had cardiovascular instability whereas only 6.7% (n=1) of the 15 patients without cranial nerve dysfunction had autonomic dysfunction (Table 5).

**Table 5:** Frequency of cardiovascular instability in patients with and without lower cranial nerve dysfunction Data has been represented has % of total, p< 0.05 has been considered as significant

Lower cranial nerve (VII, IX, X) involvement	Cardiovascular instability		Total	Chi-square	p-value
	Yes, n (%)	No, n (%)			
Absent	1 (6.7%%)	14 (93.3%)	15	5.556	0.018
Present	14 (40%)	21 (60%)	35		

In this study, 100% of the eight patients who required mechanical ventilation had evidence of cardiovascular instability. In the 42 patients who did not require mechanical ventilation, 16.7% (n=7) had cardiovascular instability and the correlation was statistically significant with a p-value of 0.00001 (Table 6).

**Table 6:** Frequency of cardiovascular instability in patients required mechanical ventilation and who did not require mechanical ventilation Data has been represented has % of total, p< 0.05 has been considered as significant

Requirement of mechanical ventilation	Cardiovascular instability		Total	Chi-square	p-value
	Yes, n (%)	No, n (%)			
No	7 (16.7%%)	35 (83.3%)	42	22.22	0.00001
Yes	8 (100%)	0 (0%)	8		

## Discussion

GBS is characterized by symmetric progressive weakness associated with hyporeflexia/areflexia and may be associated with cranial nerve palsy and/or respiratory weakness. The clinical presentation is heterogeneous with multiple variants and the severity of illness also varies. Molecular mimicry leads to antiganglioside production and subsequent complement-mediated damage and mononuclear cell infiltrate-mediated inflammation results in damage to axons or myelin. Plasma exchange and Intravenous immunoglobin (IVIg) are the evidence-based treatment options; however, 25% go on to require mechanical ventilation and 20% remain unable to walk after six months [7]. Although GBS has been classically associated with areflexia, 9% of people have retained reflexes over weak upper limbs and 2% have retained reflexes over weak legs [13]. The presence of GBS with exaggerated or preserved reflexes has especially been noted in the AMAN variant of GBS [14].

GBS produces autonomic dysfunction in 70% of patients and it may take the form of fluctuations in heart rate, blood pressure, diaphoresis, and gastrointestinal disturbances. The mortality rate is 7% among this group with autonomic disturbances and early recognition and management including prevention of triggers of autonomic storms may improve outcomes [15]. Another study published in 2022 showed a prevalence of 31% for autonomic dysfunction [16]. In the current study involving 50 patients, cardiovascular instability was present in 30% of cases, and 16% of cases required ventilation, which is consistent with the previously published studies which show an incidence of about 20% [7].

In the present study, cardiovascular instability was present in all patients who required mechanical ventilation. The incidence of cardiovascular instability was also higher in patients who had lesser onset to admission times (41.9%) compared to those with higher onset to admission times (10.5%) (p-value: 0.019). Those who had EGRIS ≥4 had a higher incidence (40.6%) of cardiovascular instability when compared to those with EGRIS ≤3 (11.1%) (p-value: 0.029). Those who had lower cranial nerve involvement had a higher incidence of cardiovascular instability (40%) as compared to those without lower cranial nerve dysfunction (6.7%) (p-value: 0.018). There is a trend of increased incidence of cardiovascular instability in those with elevated liver transaminases ≥3 ULN (50%) as compared to those with a transaminase level ≤2 ULN (26.2%), however, the correlation did not reach statistical significance (p-value: 0.178). A previous study also showed an increased prevalence of autonomic dysfunction in those with lower cranial nerve (VII, IX, X) involvement, mechanical ventilation, ICU requirement, higher EGRIS, and delirium [16].

This study has some limitations. The sample size of 50 is small compared to previous studies. There may be an institute bias since the sample population is taken from a tertiary care hospital and the clinical severity of patients admitted to this institute may not exactly mirror the severity profile of the population as a whole. Further investigation into the predictors of cardiovascular instability using appropriately powered studies with larger sample sizes and multicentric studies may help mitigate these limitations.

## Conclusions

The presence of admission EGRIS ≥4, symptom onset to admission time ≤5 days, facial palsy or bulbar palsy, and requirement of mechanical ventilation have been positively associated with the development of cardiovascular instability among patients with GBS and may warrant intensive monitoring for cardiovascular instability. Elevated liver transaminases also show a trend for an increased occurrence of cardiovascular instability; however, the correlation between elevated liver enzymes and cardiovascular instability did not reach statistical significance.
